# Novel peptides from the edible bivalve *Ruditapes decussatus* target apoptosis, autophagy, and FGF19-FGFR4 signaling in human cancer cell lines

**DOI:** 10.1038/s41598-025-20094-7

**Published:** 2025-10-01

**Authors:** Ahmed A. A. Hussein, Maha B. Salem, Mohamed ElZallat, Samah I. Ghoname, Mohamed R. Habib, Olfat Ali Hammam, Ehab El-Dabaa, Hend Okasha

**Affiliations:** 1 Department of Medical Malacology, Theodor Bilharz Research Institute, Warrak El-Hadar, Imbaba, Giza, 12411 Egypt; 2 Department of Pharmacology, Theodor Bilharz Research Institute, Warrak El-Hadar, Imbaba, Giza, 12411 Egypt; 3 Department of Immunology, Theodor Bilharz Research Institute, Warrak El-Hadar, Imbaba, Giza, 12411 Egypt; 4 Department of Pathology, Theodor Bilharz Research Institute, Warrak El-Hadar, Imbaba, Giza, 12411 Egypt; 5 Department of Biochemistry and Molecular Biology, Theodor Bilharz Research Institute, Warrak El-Hadar, Imbaba, Giza, 12411 Egypt

**Keywords:** Bivalves, Bioactive peptides, Anticancer activity, Mass spectrometry, Natural agents, Biotechnology, Cancer, Cell biology, Drug discovery

## Abstract

Bioactive peptides have recently emerged as promising candidates for cancer treatment due to their selective cytotoxicity toward cancer cells. The bivalve mollusk *Ruditapes decussatus* contains bioactive compounds that have not been thoroughly investigated for their potential anticancer properties. In this study, isolation and purification of peptide mixtures from *R. decussatus* were performed using FPLC chromatography followed by de novo sequence analysis. Using de novo peptide sequencing, a total of 135 peptides (ranging from 2681.6 to 5925.12 Da) were identified, of which 57 peptides (42%) were predicted to exhibit anticancer potential upon analysis with AntiCP 2.0, highlighting their possible therapeutic utility (Data are available via ProteomeXchange with identifier PXD067801). Additionally, fractions were tested against liver (HepG2) and colorectal (HT-29) cancer cell lines, as well as normal human hepatocytes and VERO (obtained from kidney) cells, to evaluate their cytotoxic effects. Fractions 2 and 3 showed significant anticancer activity against both cancer cell lines, while exhibiting minimal cytotoxicity toward normal cells. These fractions induced apoptosis, as evidenced by the downregulation of Bcl-2 and upregulation of caspase-3, and also activated autophagy, marked by increased Beclin-1 expression. Flow cytometry analysis revealed enhanced apoptotic cell death and G1/S phase cell cycle arrest in the treated cancer cells. Morphological analysis further confirmed the presence of apoptotic changes. Overall, the peptides derived from *R. decussatus* demonstrated the ability to induce apoptosis and cell cycle arrest in cancer cells, with a highly selective effect on colorectal carcinoma, suggesting their potential as anticancer agents for further investigation.

**Supplementary Information:**

The online version contains supplementary material available at 10.1038/s41598-025-20094-7.

## Introduction

Cancer remains a significant worldwide health concern, with approximately 19.3 million new cases and 10 million cancer-related fatalities recorded in 2020^1,2^. Hepatocellular carcinoma (HCC) and colorectal cancer (CRC) are among the most common and serious types of cancer globally, necessitating a comprehensive approach for their diagnosis, treatment, and prevention^3^. HCC ranks as the third most common cancer in men and the eighth in women worldwide^4^. Similarly, CRC is the third most diagnosed cancer overall, with men ranking third and women second. HCC and CRC develop through a multifactorial process, often initiated by carcinogenic factors that cause DNA structural changes, leading to the transformation of normal cells into cancerous ones^5^. Chemotherapy is still a mainstay of modern oncology, but among cancer survivors, its harmful side effects are a leading cause of morbidity and death^6^. Most traditional chemotherapy drugs work by targeting DNA synthesis and cell replication in tumor cells. While effective, these treatments often come with significant toxicity due to their lack of selectivity^7^.

In recent years, there has been growing interest in marine bioactive peptides owing to their promising therapeutic potential. Remarkably, during the 1980s and 1990s, over half of the drugs approved by the FDA were derived from marine organisms, highlighting the valuable role of aquatic biodiversity in pharmaceutical development^8^. Since the approval of cytarabine in 1969, numerous marine-derived compounds have been developed into anticancer agents, contributing to advancements in oncology^9^. Currently, the regulatory agencies in Europe and the United States have approved 11 marine-derived drugs, four of which—Cytosar-U, Yondelis, Halaven, and Adcetris—are specifically indicated for cancer treatment^10^.

Among marine-derived compounds, bioactive peptides have demonstrated promising anticancer potential by modulating key molecular pathways involved in tumor progression^11^. These peptides, which are naturally found in various marine organisms, including bivalves, possess unique pharmacological properties^12^. Their therapeutic versatility stems from their small size, structural adaptability, and ability to cross cell membranes, making them valuable candidates for drug development^13^. Compared to proteins or antibodies, marine peptides offer several advantages, such as ease of synthesis (e.g., cyclotide kalata B1 from sea squirts)^14^, modifiability (e.g., ziconotide, a conotoxin-derived painkiller)^15^, lower likelihood of drug-drug interactions, and reduced toxicity due to minimal accumulation in the liver and kidneys^16^. Given their chemical and biological diversity, marine peptides represent a promising avenue for developing novel anticancer therapeutics^17^. However, to clarify their modes of action and therapeutic efficacy, more investigation is needed.

Previous studies have demonstrated that marine bivalves are valuable sources of bioactive compounds with anticancer potential, as shown by the identification of novel peptides from the edible bivalve *Callista chione* with activity against hepatic and colon cancer cell lines, and by broader evidence of natural anticancer peptides from marine animal species demonstrated in various in vitro cancer models^11,18,19^. Clams yield bioactive peptides like antibacterial mytimicins (*Mytilus edulis*), antiviral venerupin (*Ruditapes philippinarum*), and antioxidant/antihypertensive peptides (*Meretrix meretrix*), demonstrating their therapeutic potentials against infection, cancer, and cardiovascular disease with favorable safety profiles^20,21^. Building on these evidences, this study aims to investigate the anticancer properties of novel peptides isolated from *R. decussatus*, focusing on their effects on apoptosis, autophagy, and the FGF19-FGFR4 signaling pathway in HepG2 and HT-29 cell lines.

## Materials and methods

### Bivalve collection and soft tissue isolation

Lake Timsah, a key aquatic ecosystem located around 76 km down to Port Said and spanning about 14 square kilometers along the Suez Canal, served as the sampling site for this study. Specimens of *R. decussatus*, a bivalve commonly consumed by residents of the Suez Canal region^22^, and selected for its potential bioactive properties, were collected from two distinct locations within the lake—Etab and the Presidential Resthouse—at depths ranging from 3 to 7 m. Viability was maintained by carefully harvesting individuals with shell lengths ranging from 2.0 to 3.0 cm and moving them right away to a flow-through aquarium system. After that, the samples were carefully transported to the Medical Malacology Department, Theodor Bilharz Research Institute for soft tissue extraction and dissection.

### Peptide extraction and purification

To guarantee protein stabilization and preservation, the soft tissues were carefully removed from the shell and submerged right away in a cold precipitation mixture that contained 24% trichloroacetic acid (TCA) and acetone, enhanced with 0.07% β-mercaptoethanol (β-ME). The tissues were further grinded using a mortar. After this, the mixture was kept on ice and vortex homogenized for a few minutes. The homogenized samples were then kept at − 20 °C for 2 h. To separate the supernatant from the pellet, at 4 °C, centrifugation was carried out for 30 min at 21,000 × *g*. The resulting pellet was collected and subjected to two washes with 10 ml of ice-cold acetone, one of which contained 0.07% β-ME. Each washing step lasted for 2 h, with the samples kept at − 20 °C throughout the process^23^. The final wash was done with 50 mM Tris base at pH 8 and the resulting pellet was dissolved in 500 µL of 50 mM Tris base at pH 9 followed by concentration using Vivaspin 20 filter devices with MWCO 3 kDa (Sartorius) to concentrate the peptides with MW ≤ 3 kDa. Peptide fractionation was performed using Fast Protein Liquid Chromatography (FPLC) system (ÄKTA Purifier 100, GE Healthcare Life Sciences, USA) equipped with a strong anion exchange QxL column. The fraction separation was monitored at 210 nm using a linear isocratic elution over 20 column volumes (CV). The mobile phases included a starting buffer of 50 mM Tris base at pH 9 and an elution buffer composed of 50 mM Tris base at pH 9 supplemented with 1 M NaCl. Fractions were collected according to absorbance at 210 nm, and their peptide concentrations were subsequently assessed using a NanoDrop 2000 spectrophotometer at the same wavelength^24^. The mass spectrometry proteomics data have been deposited to the ProteomeXchange Consortium via the PRIDE^25^ partner repository with the dataset identifier PXD067801.

### Comprehensive evaluation of peptides’ fractions (PFs): testing of cell viability, toxicity and cell morphology.

The safety profiles of three isolated PFs were evaluated using normal human hepatocytes and VERO (kidney epithelial cells from an African green monkey) cells. To evaluate anticancer effects, PFs were tested on two human cancer cell lines—HepG2 (liver carcinoma) and HT-29 (colorectal adenocarcinoma)—sourced from the Tissue Culture Unit of VACSERA (Dokki, Giza, Egypt). Both cell lines were grown in Dulbecco’s Modified Eagle Medium (DMEM; Lonza, Walkersville, MD, USA), supplemented with 10% heat-inactivated fetal bovine serum (Gibco, Life Technologies, USA), along with 1% L-glutamine, HEPES buffer, 100 U/mL penicillin, and 50 µg/mL gentamycin (Gibco, Life Technologies, USA). Cells were cultured under standard conditions at 37 °C in a humidified incubator containing 5% CO₂. Subculturing was performed biweekly, and cells were routinely passaged once they reached approximately 80% confluence.

Cytotoxic effects of the PFs were assessed using the MTT assay (3-(4,5-dimethylthiazol-2-yl)-2,5-diphenyltetrazolium bromide). Cells were plated in 96-well plates at a density of 1 × 10^4^ cells per well and allowed to adhere overnight. The culture medium was then replaced with either a vehicle control or varying concentrations of PFs (ranging from 2.5 to 80 µg/mL), followed by a 24-h incubation period. After treatment, an MTT solution (10% of 2 mg/mL; Sigma-Aldrich, USA) was added to each well and incubated for 4 h at 37 °C in the absence of light. The resulting formazan crystals were solubilized by adding 100 µL of dimethyl sulfoxide (DMSO; Sigma-Aldrich, USA) and gently shaking for 5 min. Absorbance was recorded at 540 nm using a BioTek microplate reader (Agilent Technologies, USA). Cell viability was determined using the equation: Viability (%) = [1 − (ODₜ/OD_c_)] × 100, where ODₜ and OD_c_ represent the absorbance of treated and control wells, respectively. Each treatment was conducted in triplicate, and experiments were independently repeated three times to determine the IC₅₀ values of the tested fractions. Finally, the cell morphology of treated cells was assessed by culturing cells in 24-well plates and treating them with DMSO or different concentrations of PFs for 24 h. Cell morphology was compared to untreated cells using an inverted light microscope (Optika, Italy) and photographs were captured.

### Sequencing of collected fractions using mass spectrometry

An Orbitrap Exploris 480 Mass Spectrometer (Thermo Scientific) was used to evaluate the peptide fractions that were obtained from the chromatography. The samples were first subjected to UHPLC separation, employing Buffer A (0.05% formic acid in water) and Buffer B (0.05% formic acid in acetonitrile). Injection was performed on a C18 trapping column (5 µm particle size, Thermo Scientific), followed by gradient elution. Initially, the mobile phase consisted of 98% Buffer A (0.1% formic acid) and 2% Buffer B (80% acetonitrile, 0.05% formic acid) for 2 min at a flow rate of 250 µL/min. Buffer B concentration was then increased to 7.5% over 1 min, and further raised linearly from 7.5 to 37.5% during the subsequent 120 min. To re-equilibrate the column, the concentration of Buffer B was increased to 42.5% over 3 min, followed by a ramp-up to 99% for 6 min, before returning to 2%^26^. The mass spectrometer operated with a full MS scan resolution of 60,000 across an m/z range of 200 to 2,000, while data-dependent MS/MS scans were conducted at a resolution of 120,000 with 30% HCD collision energy.

### Data analysis for sequence identification

As shown in Table 1, the protein database (www.uniprot.org) does not contain the full proteome of *R. decussatus*; rather, only sequences for select proteins were available. For peptide analysis, Biopharma Finder 4.0 (Thermo Scientific) software was used, and the novor.cloud web tool for proteomics mass spectrometry data analysis (https://app.novor.cloud/) was used to execute de novo sequencing.

**Table 1:** Proteins of *R. decussatus* clam identified through UniProt database.

Entry name	Protein names	Gene names	Length (AA)
A0A0D3MAZ9_9BIVA	Cytochrome c oxidase subunit 1 (EC 7.1.1.9)	COI	203
A0A0D3MB07_9BIVA			203
A0A0D3MB09_9BIVA			203
A0A0D3MB65_9BIVA			203
A0A0D3MB71_9BIVA			183
A0A0D3MB82_9BIVA			183
A0A0D3MBD5_9BIVA			183
A0A0K1DB69_9BIVA			219
A0A142BK74_9BIVA			126
A0A1L2D5N8_9BIVA			115
A0A1L2D5P1_9BIVA			115
A0A1L2D5P4_9BIVA			115
A0A1L2D5P5_9BIVA			115
A0A1L2D5P6_9BIVA			115
A0A1L2D5P7_9BIVA			115
A0A1L2D5P8_9BIVA			115
A0A1L2D5P9_9BIVA			115
A0A1L2D5Q3_9BIVA			115
A0A1L2D5Q5_9BIVA			115
A0A1L2D5Q6_9BIVA			115
A0A219LV50_9BIVA		COX1	571
A0A3G2BC06_9BIVA		COI	200
A0A6M3YVR6_RUDPH		COX1	189
A0A6M3YVT9_9BIVA			210
A0A6M3YVU4_RUDPH			189
A0A6M3YVZ9_9BIVA			210
A0A6M3YW47_RUDPH			189
A0A6M3YWE7_RUDPH			189
A0A6M3Z0D7_9BIVA			210
H9XNZ7_9BIVA		COI	218
L7QK56_9BIVA			187
Q0H6X2_9BIVA		COX1	219
Q0QBP0_9BIVA		COI	190
U5I8Q0_9BIVA			143
U5I9Z6_9BIVA			143
V9P7E2_9BIVA		COX1	297
V9P7F5_9BIVA			302
V9P7F6_9BIVA			287
V9P7G9_9BIVA			301
V9P7H0_9BIVA			280
V9P7I3_9BIVA			296
V9P7I6_9BIVA			277
V9P7Y6_9BIVA			299
V9P7Y9_9BIVA			290
A0A067XHZ5_9BIVA	Ferritin (EC 1.16.3.1)		217
A0A067XI00_9BIVA			232
A0A067XI81_9BIVA			170
A0A067XIK1_9BIVA			171
A0A219LUV1_9BIVA	NADH-ubiquinone reductase (H( +)-translocating) (EC 7.1.1.2)	ND2	339
A0A219LUV6_9BIVA	Cytochrome c oxidase subunit 2	COX2	423
A0A219LUV7_9BIVA	Cytochrome b	CYTB	407
A0A219LUV8_9BIVA	NADH-ubiquinone reductase (H( +)-translocating) (EC 7.1.1.2) (NADH dehydrogenase subunit 5)	ND5	545
A0A219LUV9_9BIVA	NADH-ubiquinone oxidoreductase chain 3 (EC 7.1.1.2)	ND3	135
A0A219LV19_9BIVA	Cytochrome c oxidase subunit 3	COX3	321
A0A219LV72_9BIVA	NADH-ubiquinone oxidoreductase chain 4 (EC 7.1.1.2)	ND4	446
A0A219LVD8_9BIVA	ATP synthase subunit a	ATP6	245
A0A219LVW1_9BIVA	NADH-ubiquinone oxidoreductase chain 1 (EC 7.1.1.2)	ND1	305
B3FEA8_9BIVA	Histone H1	H1	191
B3FEA9_9BIVA	Histone H2A	H2A	125
B3FEB0_9BIVA	Histone H2B	H2B	124
B3FEB1_9BIVA	Histone H3	H3	136
B3FEB2_9BIVA	Histone H4	H4	103
B3FRR7_9BIVA	Ferritin (EC 1.16.3.1)		129
C4N894_9BIVA	Complement C2 (EC 3.4.21.43)		697
Q0QBK1_9BIVA	Histone H3	H3	109
A0A219LUV5_9BIVA	ATP synthase subunit 8	ATP8	41
A0A219LUW0_9BIVA	NADH dehydrogenase subunit 4L	ND4L	90
A0A219LVW6_9BIVA	NADH dehydrogenase subunit 6	ND6	164
A7LBN4_9BIVA	Metallothionein	MT	72
B3FRR6_9BIVA	Heat shock protein 70		174
Q6SQL4_9BIVA	Actin		208
Q6SQL5_9BIVA	Actin		208
Q6U6G9_9BIVA	Cu/Zn-superoxide dismutase (EC 1.15.1.1)		131
Q9N9H1_9BIVA	Metallothionein	Mt	59
U5I9K6_9BIVA	Enoyl coenzyme A hydratase	Ech	39

### Peptide analysis via de novo sequencing and AntiCP 2.0

The antiCP 2.0 web server (https://webs.iiitd.edu.in/raghava/anticp2/) was used to examine the peptide sequences that were obtained from novor.cloud. This platform employs a strict antiCP prediction model that assigns a score indicating the likelihood of each peptide exhibiting anticancer properties. The computational scores range between 0 and 1, where peptides with higher values are predicted to have stronger anticancer activity. Peptides that functioned as anticancer peptides had a value above the threshold (0.5). There have also been reports of physicochemical characteristics such as charge, molecular weight (MW), amphipathicity, hydrophilicity, hydropathicity, sidebulk, hydrophobicity, and isoelectric point (pI). These characteristics provide a good understanding of each peptide’s kind as well as its functional and chemical characteristics^27^.

### Quantitative detection of apoptotic and autophagy markers

Following treatment with the IC_50_ concentrations for those peptide samples, total RNA was extracted from HepG2 and HT-29 cells using the Trizol reagent (Biovision Co. LTD, Korea) following the manufacturer’s guidelines. The RevertAidTM First Strand cDNA Synthesis Kit (Thermo Scientific, Cat. No. K1621) was then used to create complementary DNA (cDNA) from the extracted RNA in compliance with the instructions provided. protocol. The specific primer sequences used for amplifying Beclin-1, Bcl-2, caspase-3, FGFR4, and FGF19 (F-19) genes are detailed in Table 2. The housekeeping gene used for normalization was GAPDH.

**Table 2:** Primer sequences for quantitative real-time PCR analysis.

Target gene (s)	Primer sequence	Accession no
Beclin-1	Forward primer: 5′-GAGAGACCCAGGAGGAAG-3′	XM_017025264.3
	Reverse primer: 5′-GGCCCGACATGATGTCAA-3′	
Bcl-2	Forward primer: 5′-CCTGGCTGTCTCTGAAGACC-3′	NM_016993.2
	Reverse primer: 5′-CTCACTTGTGGCCCAGGTAT-3′	
Caspase-3	Forward primer: 5′-TGCATACTCCACAGCACCTG-3′	XM_054350958.1
	Reverse primer: 5′-TCTGTTGCCACCTTTCGGTT-3′	
FGFR4	Forward primer: 5′-CACTGGTACAAGGAGGGCAG-3′	NM_001354984.2
	Reverse primer: 5′-ATCGTTGCTGGAGGTCAAGG-3′	
FGF19	Forward primer: 5′-TGTGTGGTGGTCCACGTATG-3′	NM_005117.3
	Reverse primer: 5′-CGGATCTCCTCCTCGAAAGC-3′	
GAPDH	Forward primer: 5′-CCCATCACCATCTTCCAGGAGC-3′	NM_001357943.2
	Reverse primer: 5′-CCAGTGAGCTTCCCGTTCAGC-3′	

### Evaluation of fractions’ treatment impacts on cancer cells through flow cytometry

For further analysis, a confluent sheet of HepG2 cells was used for flow cytometry. The bioactivity of cells treated with IC_50_ of fraction (2) and fraction (3) were compared with untreated HepG2 cells. After treatment, the two groups were made to stand for a certain period to allow cells response. Following post-incubation, the cells derived from both groups were trypsinized, pelleted, washed, and resuspended in an equal volume of 1 × PBS to obtain single-cell suspension. The cells were washed several times and fixed in cold ethanol and the samples were treated with ribonuclease to ensure that only the DNA was stained. Finally, the cell samples were stained with propidium iodide (PI) to differentiate between the G0/G1, S, and G2/M stages of the cell cycle. The flow cytometry data were collected and analyzed using computer software (flowjo v.10) to obtain the proportion of cells.

### Cytopathological examination of treated cells

HepG2 and HT-29 (1 × 10^4^) cells were trypsinized and subjected to the IC_50_ of fractions 2 and 3, followed by a pH = 7.4 PBS wash and collection in a tube. The samples were centrifuged for 15 min at a rate of 1,200–1,500 rpm. On glass slides, the cell pellet was spread out, and the slides were fixed in 95% ethanol for a whole day. On the slides, (H&E) was applied.

### Statistical analysis

Data are presented as the mean ± standard deviation (SD). Statistical analyses were conducted using GraphPad Prism version 6 (GraphPad Software Inc., San Diego, CA, USA). Comparisons between treatment groups and the control were made using Student’s t-test. Levels of statistical significance were indicated by asterisks: * for *p* < 0.05, ** for *p* < 0.01, and *** for *p* < 0.001. In addition, dose–response curves were generated for each treatment to calculate the IC_50_ (half-maximal inhibitory concentration).

## Results

### Peptide extraction and purification

**Fig. 1:**
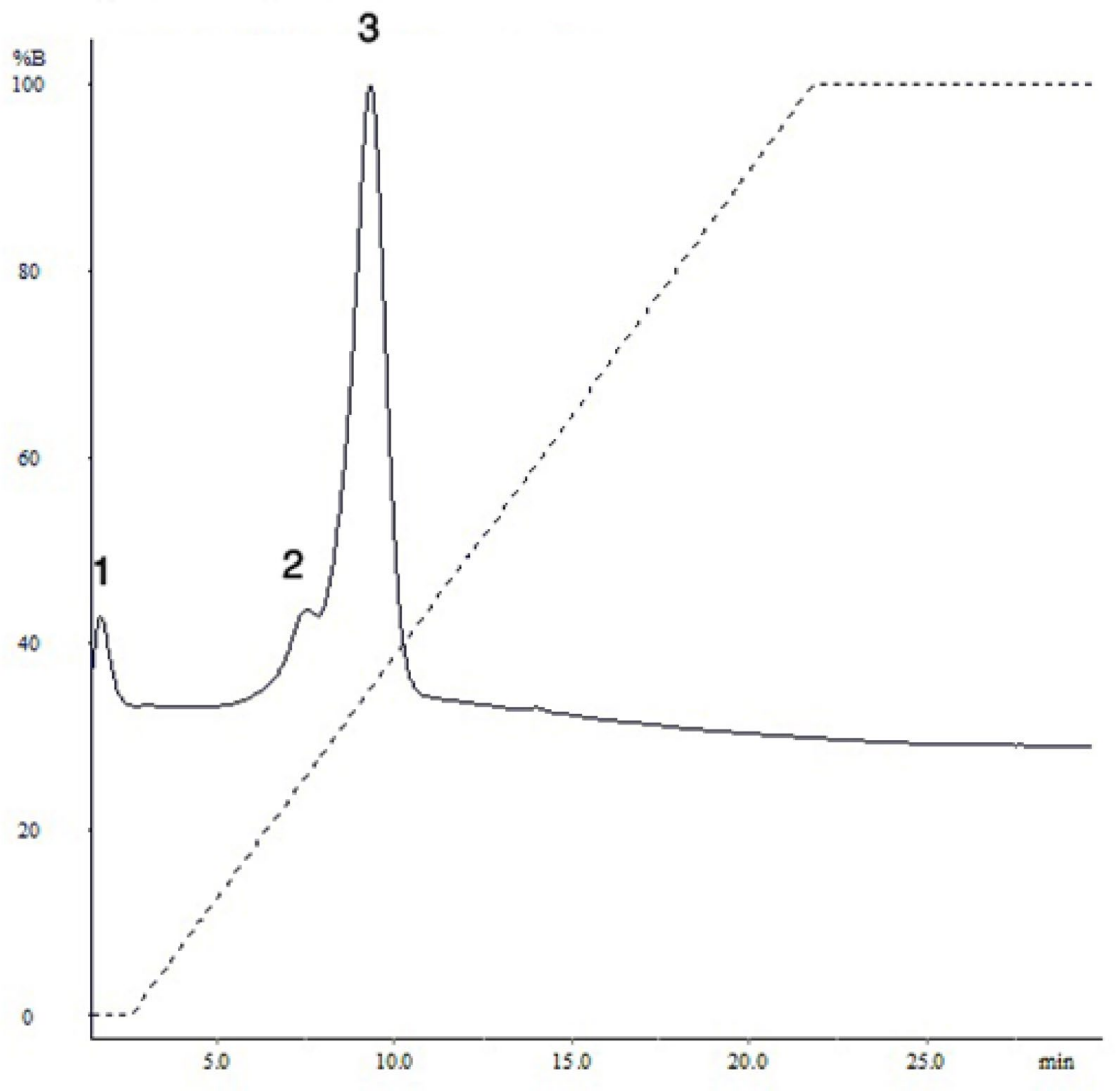
The chromatogram of the fractionation of purified peptides mixture isolated from *R. decussatus* using a QxL strong anion exchange column. The X-axis represents the isocratic gradient in time and the Y-axis represents the absorbance; mAU, 210 nm.

A peptide mixture with a concentration of 5 mg/mL (determined by absorbance at 210 nm) was loaded onto the column for purification. Peptide separation during isocratic elution was based on charge differences. As illustrated in the chromatogram (Fig. 1), three distinct peaks were detected: one during the washing step at a retention time of 0.16 min, and two during the elution phase at 7.14 and 7.65 min, respectively. The resulting fractions were labeled 1, 2, and 3, each representing peptides with similar charge properties. Concentrations of the collected fractions, measured at 210 nm, were 0.67 ± 0.16, 46 ± 2, and 140 ± 4.4 µg/mL, respectively. Fraction 1 was excluded from further analysis due to its low concentration (< 1 µg/mL), while the remaining fractions underwent mass spectrometry to determine peptide sequences.

### Sequencing of collected fractions using mass spectrometry and de novo analysis

Peptide analysis was performed on fraction (2) using Biopharma Finder 4.0 software (Thermo Scientific) with reference to the identified *R. decussatus* clam proteins from the UniProt database. Results showed that only four peptides had been recognized to be part of a sequence in *R. decussatus* (Table 3). The results obtained showed that all defined peptides showed nonspecific modification. In addition, the expanded retention time from 1.33 to 17.23 min reflects the accuracy of chromatographic separation before mass spectrometry. Experimental average masses closely matched their respective theoretical monoisotopic masses, demonstrating the reliability of the mass spectrometry results. The peptides were mapped to specific protein entries, confirming that the obtained peptide sequences are annotated to the Cytochrome c oxidase subunit 1 protein. In the case of fraction (3), no data was obtained from the Biopharma Finder 4.0 software; hence, peptide analysis was performed using novor.cloud web tool (https://app.novor.cloud) concerning the SwissProt database. A sum of 135 peptides (from 5925.12 to 2681.6 Da) was recognized using de novo peptide sequencing. Analysis of anticancer potentiality of the obtained peptides using AntiCP 2.0 revealed that 57 peptides had anticancer capability; the mean anticancer score of anticancer peptides was calculated to be 0.62 ± 0.09. Peptide Pep25 with the sequence (KLAHRRRSKPKKWWWQGARNWWRWWWRRRFF), revealed the most promising anticancer score (0.83). The peptide mixture was more hydrophilic with a value equal to 0.12 ± 0.6 and tends to be strongly cationic with 3.9 ± 5.9 net charge and pI equal to 8.7 ± 2.98 (Table S1, supplementary).

**Table 3:** Analysis of peptides in fraction (2) using Biopharma Finder 4.0

Peptide sequence	Modification	elta (ppm)	RT	M/Z	Charge state	Avg mass exp	Mono mass theo	Protein entry name
YVLS	Nonspecific	− 8.45	1.33	481.262	1	480.43	480.2584	V9P7G9|
LVIPDMAFPRMNN	Nonspecific	− 77.24	1.59	1518.711	1	1517.7	1517.8204	V9P7F6
VLS	Nonspecific	− 1.26	1.75	318.202	1	317.19	317.1951	V9P7G9
AFPRMNNASFWFL	Nonspecific	− 6.93	17.23	401.217	4	1600.84	1600.8478	V9P7I6

### Comprehensive evaluation of PFs: testing of cell viability, toxicity

**Fig. 2:**
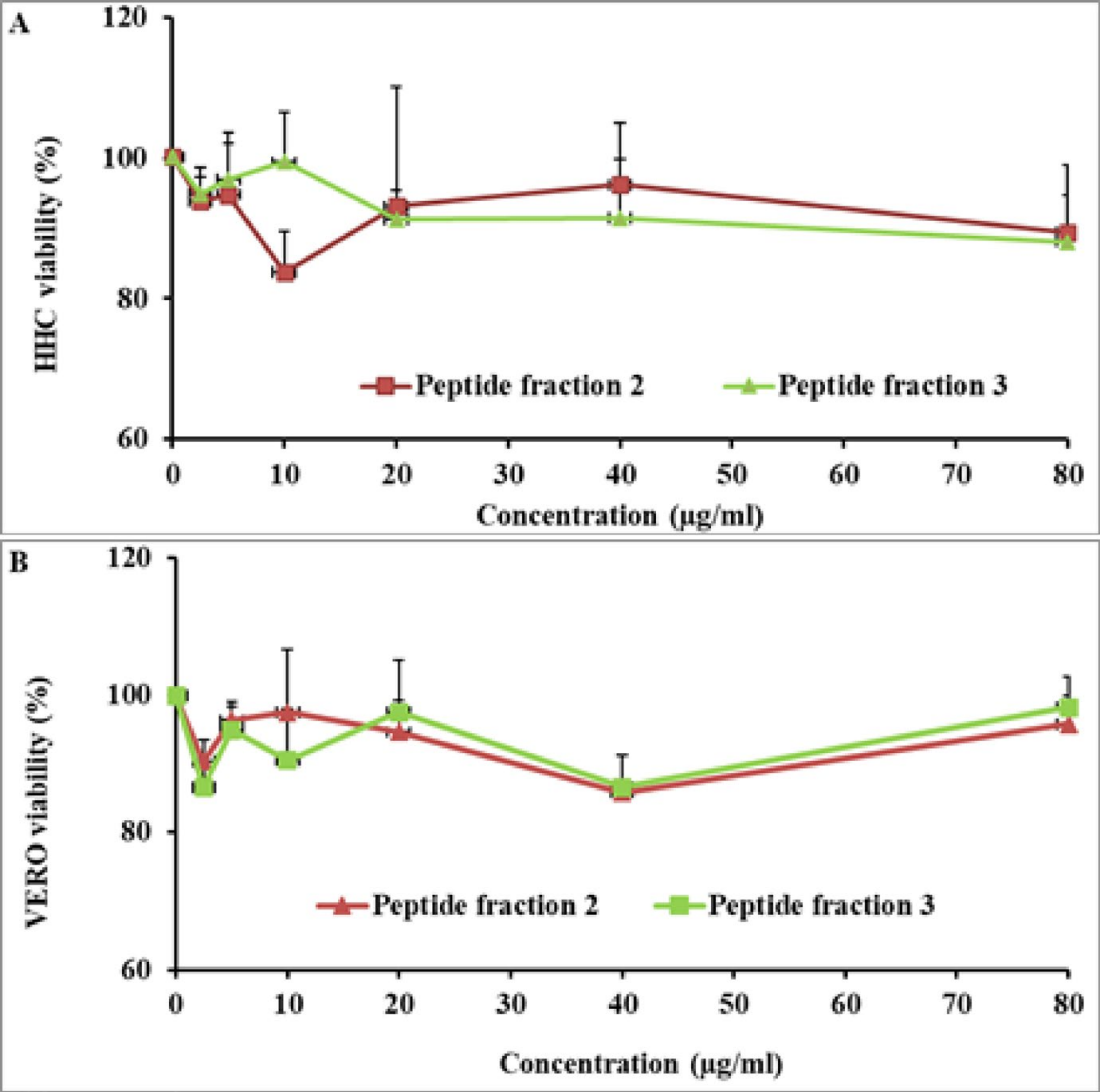
Safety profile of *R. decussatus* isolated peptides on (**A**) HHC and (**B**) VERO cell lines. Values as presented as mean ± SD.

**Fig. 3:**
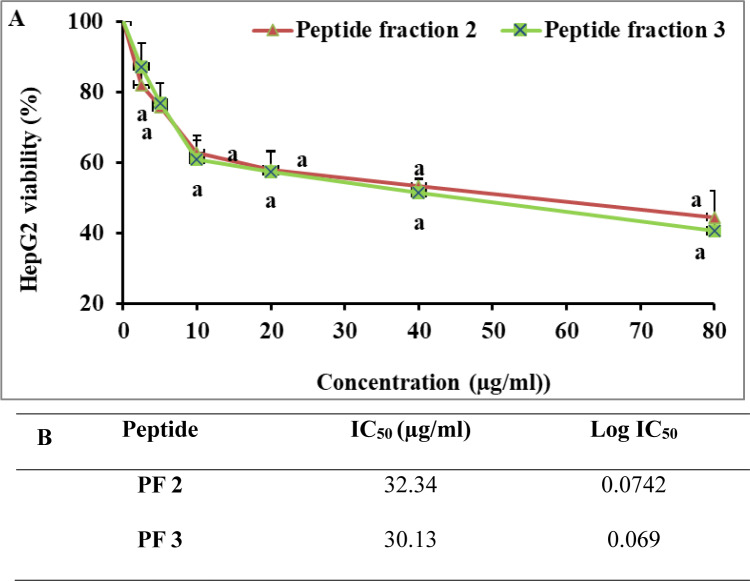
Cytotoxic effect of *R. decussatus* isolated peptides on (**A**) HepG2 and (**B**) IC_50_. Values presented as mean ± SD*.* a indicates a significant difference compared to the control (*p* < 0.05).

**Fig. 4:**
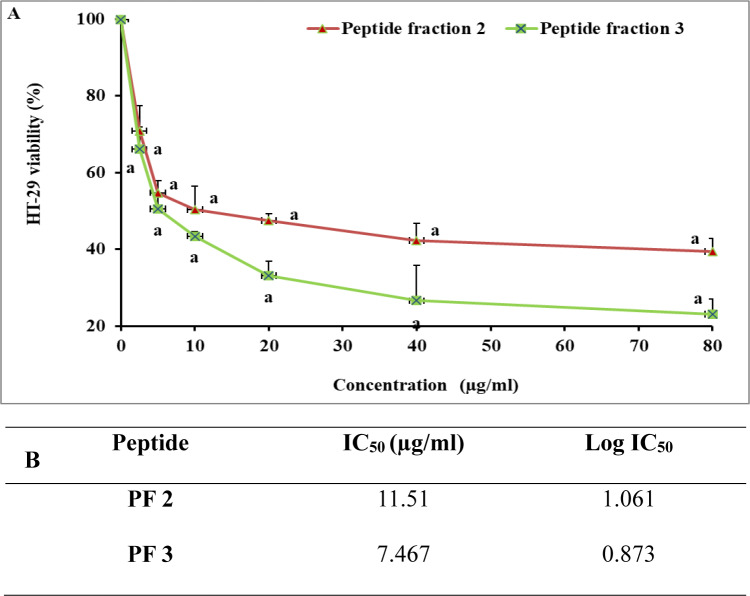
Cytotoxic effect of *R. decussatus* isolated peptides on (**A**) HT-29 and (**B**) IC_50_. Values presented as mean ± SD. a indicates a significant difference compared to the control (*p* < 0.05).

Figures 2, 3, and 4 illustrate the cytotoxic and safety assessment of two bioactive peptide fractions, PF 2 and PF 3, purified from *R. decussatus*. These fractions were evaluated for their effects on both normal cell lines—specifically human hepatocytes and VERO cells—and cancerous cell lines including HepG2 and HT-29. Importantly, PF 2 and PF 3 exerted a neglected harmful influence on the viability of the normal cells, indicating a high level of safety and selective cytotoxicity.

In terms of anticancer potential, both PF 2 and PF 3 demonstrated significant inhibitory effects on HepG2 cells, with IC_50_ values calculated at 32.34 µg/mL and 30.13 µg/mL, respectively. Although their efficacy was comparable in liver cancer cells, their impact on colorectal cancer cells was more pronounced. PF 2 inhibited HT-29 cell viability with an IC_50_ of 11.51 µg/mL, while PF 3 showed even stronger activity, reducing cell viability with an IC_50_ of 7.467 µg/mL.

### Quantitative detection of apoptotic and autophagy markers

**Fig. 5:**
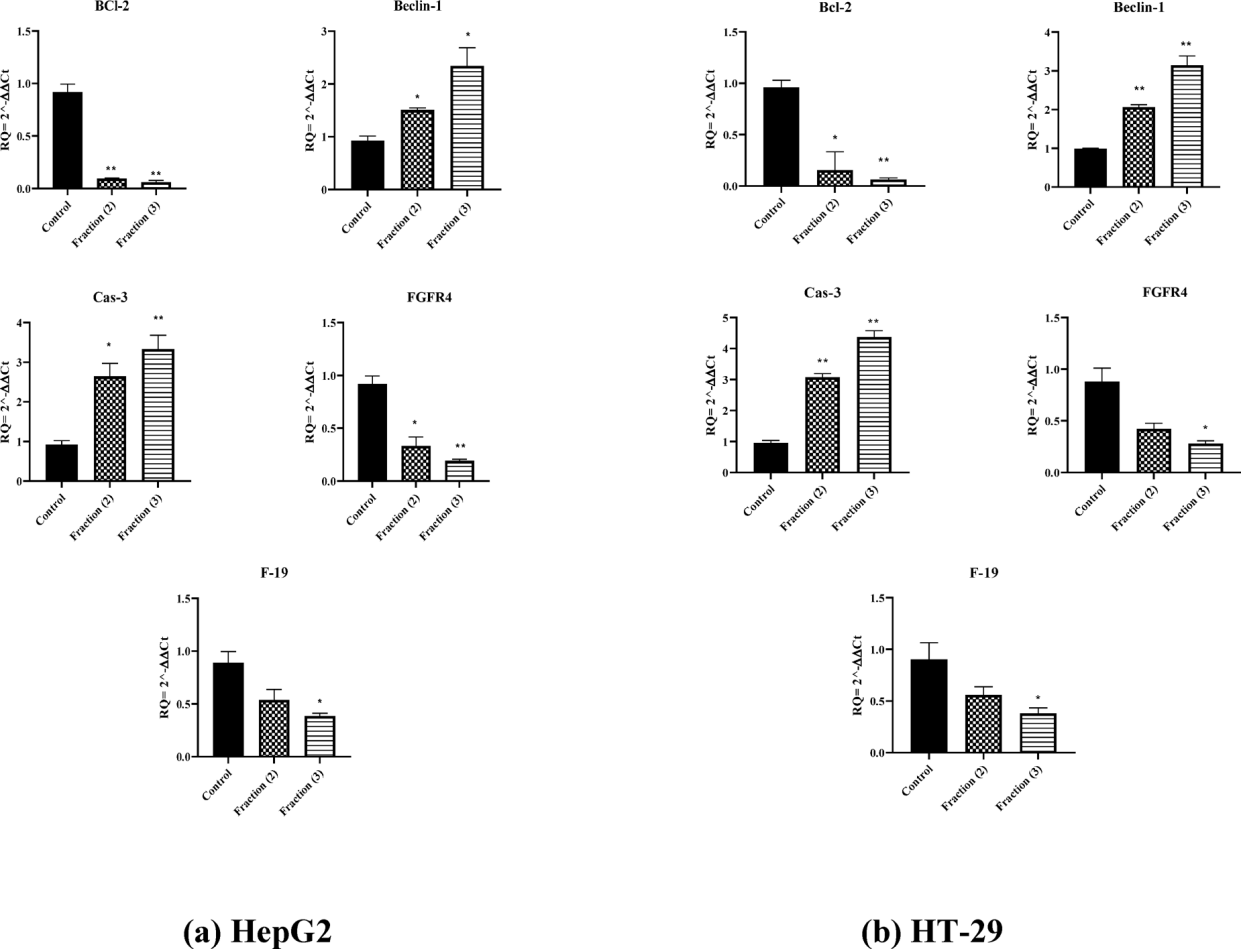
Evaluation of apoptotic and autophagy biomarkers in cancer cells under the action of purified PFs. The relative expression of important genes concerned with apoptosis and autophagy in both HepG2 (**a**) and HT-29 (**b**) cells after treatment with fractions (2) and (3) compared to untreated cells (control). (*) *p*-value < 0.05, (**) *p*-value < 0.01.

The anticancer efficacy of the two purified PFs was further assessed using HepG2 and HT-29 cells through modulation of the apoptotic and autophagy biomarkers such as Bcl-2, caspase-3, FGFR4, F-19 and Beclin-1. In HepG2 cells, the two PFs similarly caused a decrease in the levels of Bcl-2 which is associated with the reduced signaling pathway of cell survival and activation of apoptotic pathways. This was accompanied by a considerable increase in Cas-3 levels indicating that apoptotic execution phase has been triggered. Furthermore, decreased amounts of FGFR4 and F-19 were observed suggesting that the treatment affects cancer progressive markers. The same patterns occurred in HT-29 cells where the fractions caused a reduction in Bcl-2, FGFR4, and F-19 levels proving the implementation of the peptide’s pro-apoptotic and anticancer properties. Higher levels of caspase-3 indicated the enhanced apoptosis while elevated Beclin-1 levels therein suggested that the autophagy complements the processes that lead to anticancer effects. As shown in Fig. 5, the PFs could be potential anticancer agents that might interfere with the progression of cancer cells.

### Evaluation of fractions’ treatment impacts on cancer cells through flow cytometry

####  Cell apoptosis analysis

The flow cytometric analysis revealed a concentration-dependent increase in apoptosis in HT-29 and HepG2 cell lines following treatment with fraction (2) and fraction (3). After 72 h, both fractions significantly elevated early and late apoptotic cell populations, while reducing the percentage of viable cells compared to untreated controls. In HepG2 cells, the control group exhibited minimal cell death (1.73% total) and necrosis (1.28%). Treatment with fraction (2) induced a total cell death rate of 39.11%, with 9.96% early apoptosis and 24.52% late apoptosis, whereas fraction (3) resulted in 28.91% total cell death, with 5.88% early apoptosis and 19.27% late apoptosis. Similarly, in HT-29 cells, control samples showed negligible cell death (1.94% total) and necrosis (1.32%). Treatment with fraction (2) led to a total cell death rate of 31.88%, with 6.84% early apoptosis and 21.16% late apoptosis, while fraction (3) induced 27.31% total cell death, with 7.55% early apoptosis and 5.15% late apoptosis.

**Fig. 6:**
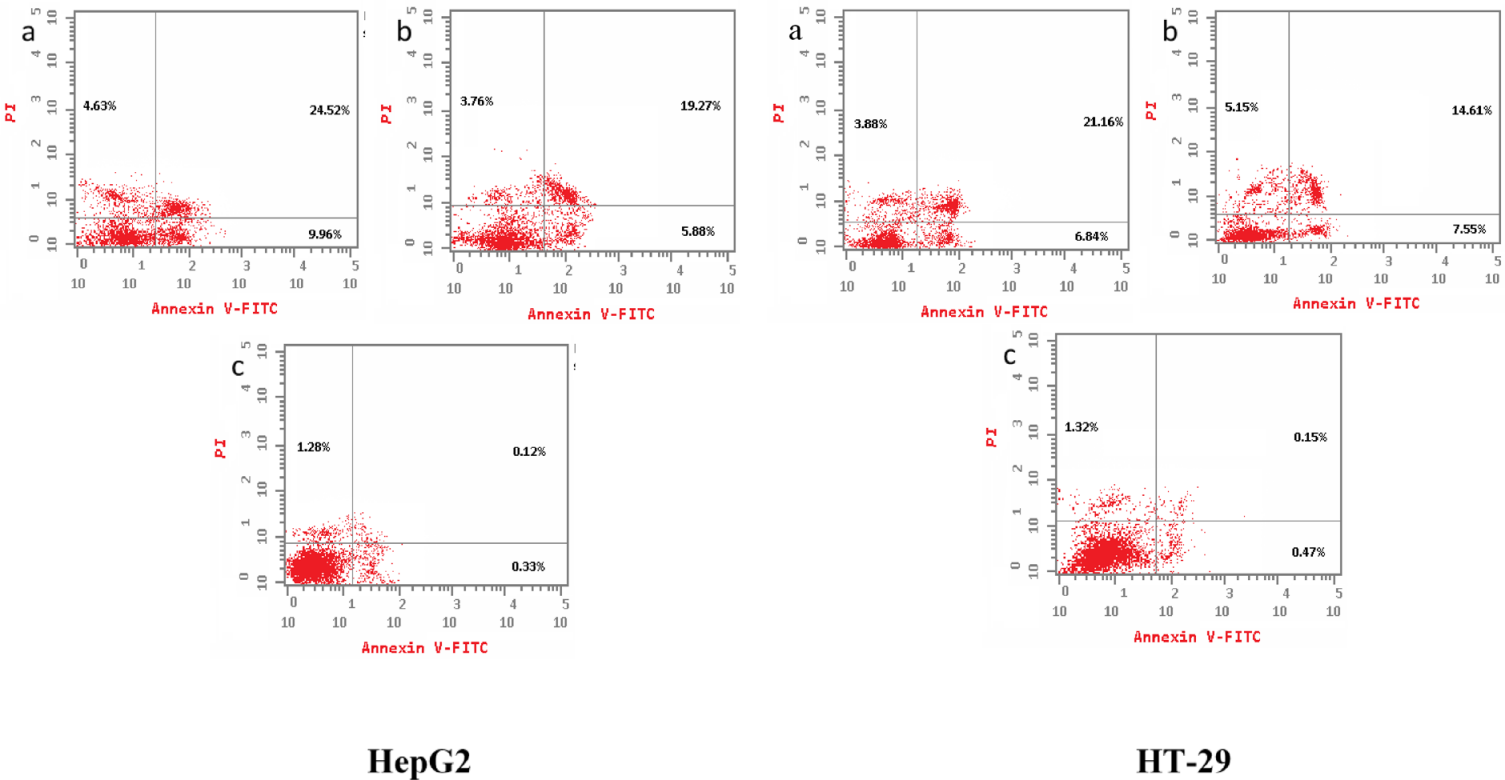
Flow cytometric evaluation of cell death in HepG2 and HT-29 cells following exposure to bioactive peptide fractions. Using FlowJo software, dot plots were generated based on dual staining with Annexin V-FITC and propidium iodide (PI) to distinguish different cell populations. Panels (**a**) and (**b**) correspond to cells treated with peptide fractions 2 and 3, respectively, while panel (**c**) depicts untreated controls. The analysis separates cell populations into four distinct groups: viable (negative for both Annexin V and PI), early apoptotic (Annexin V-positive, PI-negative), late apoptotic (positive for both markers), and necrotic (PI-positive, Annexin V-negative). This approach provides insight into the mode of cell death induced by each peptide treatment.

Comparative analysis indicated that fraction (2) exhibited a stronger apoptogenic effect in both cell lines, inducing higher levels of both early and late apoptosis compared to fraction (3). This suggests that fraction (2) may possess greater pro-apoptotic potential in HepG2 and HT-29 cells. The observed increase in apoptosis following treatment highlights the potential anticancer activity of these purified fractions, which warrants further investigation into their mechanisms of action. (Fig. 6 and Table 4).

**Table 4:** Effect of incubation with fractions (2) and (3), for 72 h on cell apoptosis in HepG2 and HT-29 cell lines.

	HepG2				HT-29			
Protein	Total	Apoptosis%		Necrosis%	Total	Apoptosis%		Necrosis%
		Early	Late			Early	Late	
Fraction (2)	39.11	9.96	24.52	4.63	31.88	6.84	21.16	3.88
Fraction (3)	28.91	5.88	19.27	3.76	27.31	7.55	14.61	5.15
Control	1.73	0.33	0.12	1.28	1.94	0.47	0.15	1.32

#### Cell cycle analysis

**Fig. 7:**
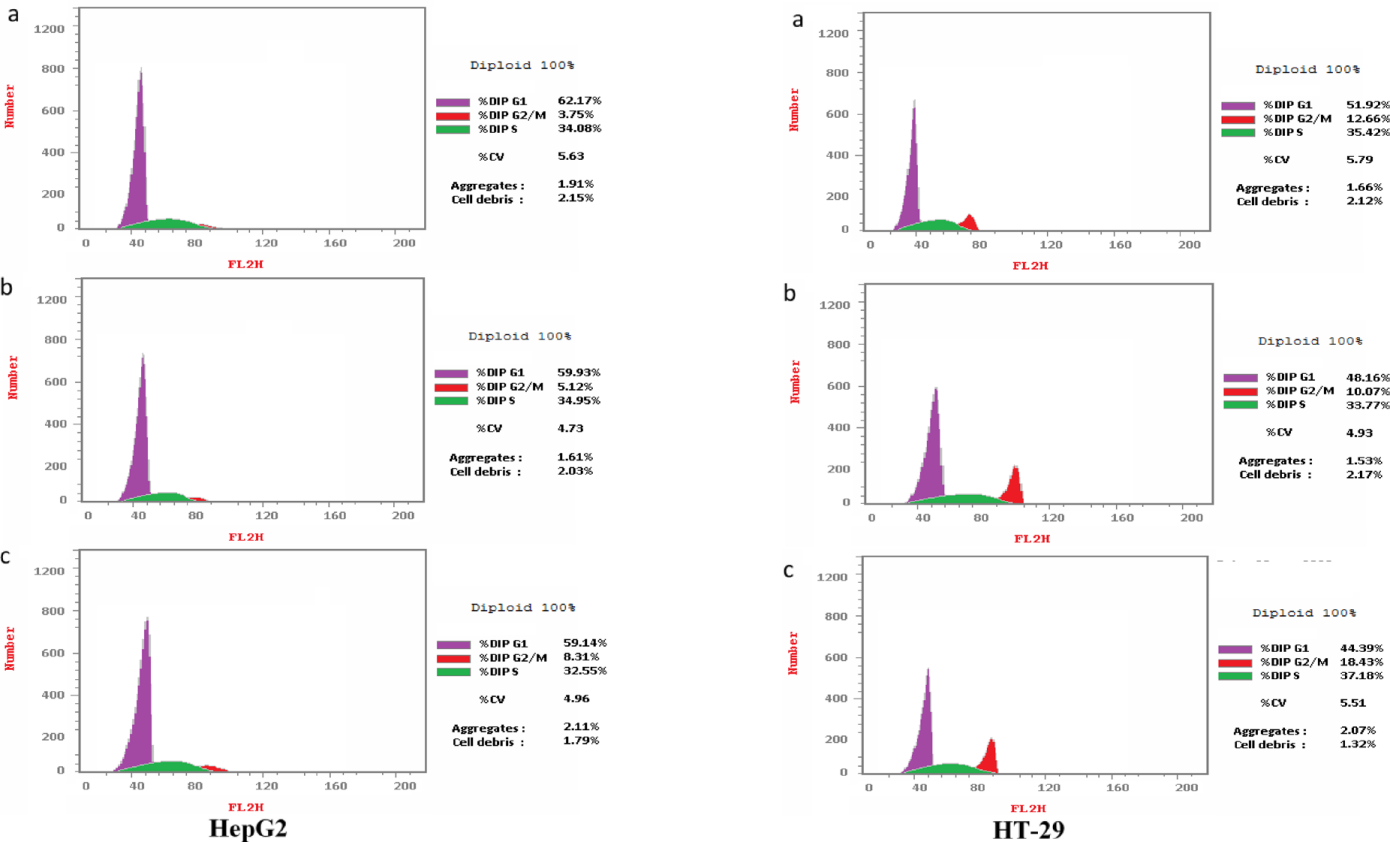
Cell cycle distribution of HT-29 and HepG2 cells following treatment with fractions (2) and (3). Flow cytometry analysis showing the effects of (**a**) fraction (2), (**b**) fraction (3), and (**c**) control on cell cycle progression after 72 h of treatment. HT-29 cells exhibited G1/S phase arrest, while HepG2 cells showed a slight increase in the G1 phase.

As illustrated in Fig. 7, treatment with fractions (2) and (3) for 72 h led to a notable increase in the percentage of HT-29 cells in the G1/S phase, suggesting a disruption in cell cycle progression. Similarly, in HepG2 cells, a slight increase in the percentage of cells in the G1 phase was observed following treatment with both fractions, indicating a potential delay in cell cycle transition. These findings suggest that the tested fractions may induce cell cycle arrest, thereby inhibiting uncontrolled proliferation in both cancer cell lines.

### Morphological evaluation of fractions’ treatment impacts on cancer cells

**Fig. 8:**
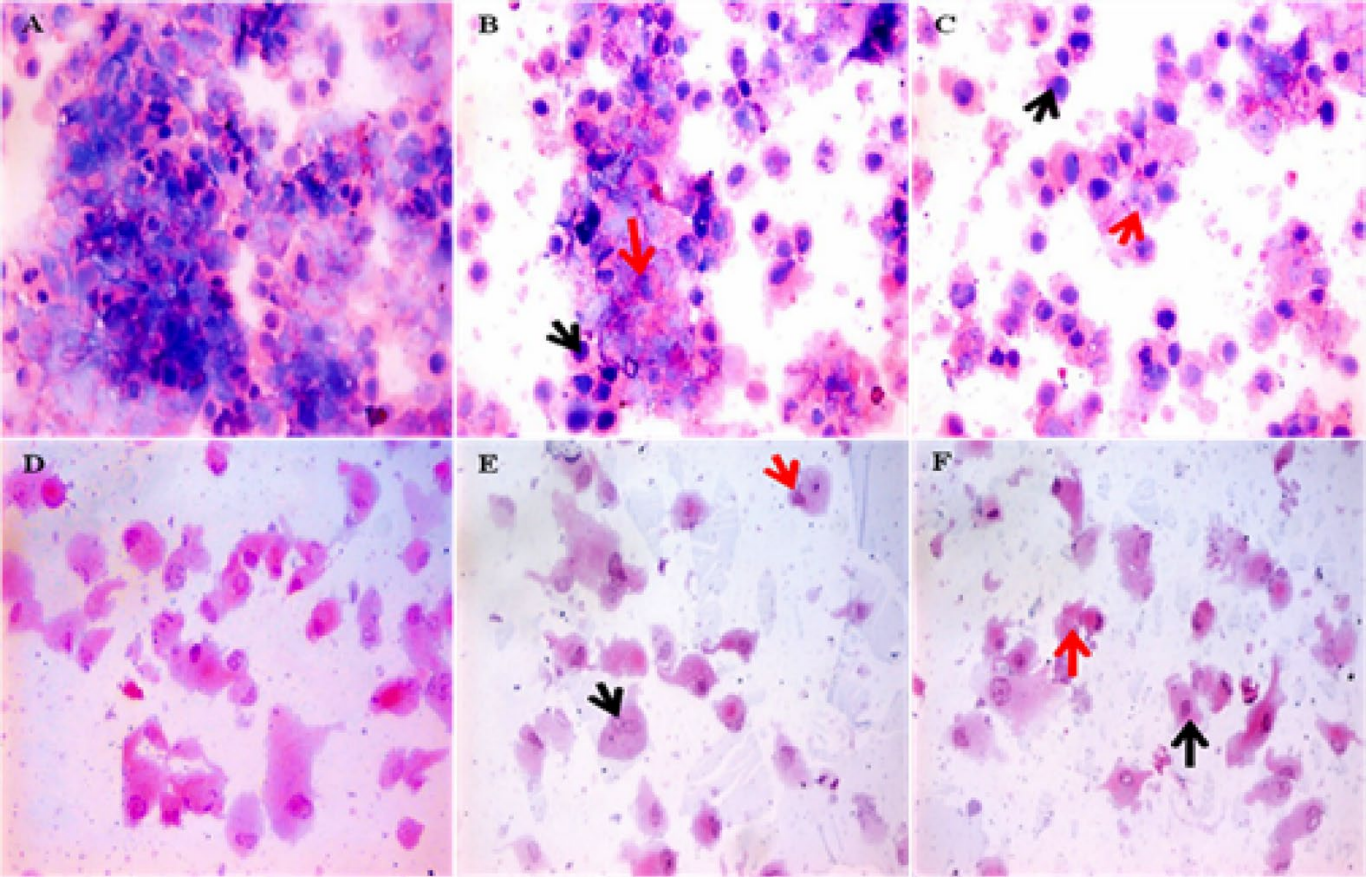
Cytospin smear stained by H & E of (**A**) control untreated HepG2, (**B**) HepG2 after exposure IC_50_ of bioactive peptides fraction 2, (**C**) HepG2 after exposure IC_50_ of bioactive peptides fraction 3, (**D**) control untreated HT-29, (**E**) HT-29 after exposure IC_50_ of bioactive peptides fraction 2, and (**F**) HT-29 after exposure IC_50_ of bioactive peptides fraction 3. Black arrow indicates nucleocytoplasmic ratio. Red arrow indicates the scattered apoptotic and degenerative changes (400x).

Treatment of HepG2 and HT-29 cells with bioactive peptide fractions 2 and 3 at their IC_50_ levels led to notable morphological alterations. As illustrated in Fig. 8, the untreated HepG2 cells predominantly displayed features typical of neoplastic hepatocytes, including clustered arrangements with prominent nuclear enlargement and elevated nucleocytoplasmic ratios. Similarly, control HT-29 cells showed abundant malignant epithelial cells marked by large nuclei and dense cytoplasmic content. Following exposure to PFs 2 and 3, HepG2 cells exhibited a more dispersed pattern, with hepatocytes displaying centrally located nuclei and evidence of apoptotic and degenerative features. In HT-29 cells, peptide treatment resulted in a reduced presence of malignant morphology, with cells showing centrally placed nuclei and moderate signs of apoptosis and degeneration, suggesting cytotoxic impact of the peptide fractions.

## Discussion

Cancer-selective anticancer peptides or anticancer peptides (ACPs) have attracted much interest as therapeutic agents because of their ability to confer deadly damage on cancer cells with minimal or no effect on normal cells^28^. These chemical messengers are generally small peptides with lengths ranging from 5 up to 50 amino acid residues^29^. It is often cationic and amphiphilic, which is good for facilitating interaction with the negatively charged outer membrane of cancer cells^30^. Marine products claim to be a reservoir of bioactive peptides; different groups of marine organisms such as sponges, cnidarians, mollusks, annelids, arthropods, echinoderms, and chordates, contain anticancer peptides that can selectively kill cancer cells^31^. These peptides affect intracellular homeostasis and act as angiogenesis inhibitors, cancer cell drug resistance reducers, metastasis inhibitors, immune system stimulants, cell differentiation promoters, and apoptosis activators^32^. Librizzi^33^ concluded that almost 100 marine peptides that exhibit anti-tumor properties have been identified, of which more than 90% of these peptides induce cancer cell apoptosis by activating caspase-dependent pathways, by causing down-regulation of anti-apoptotic factors, and by increasing intracellular oxidative stress in cancer cells.

In this study, peptides were extracted and purified from the bivalve *R. decussatus* to administer and isolate compounds that have anticancer activity. The findings suggested that the obtained PFs after fractionation, especially fractions 2 and 3, exhibit efficient cytotoxic properties against HepG2 and HT-29 cancer cell lines as well as low toxicity against normal cell lines. Therefore, these peptides represent promising candidates for selective anticancer therapy. In addition, the sequencing results indicated that some of the peptides in these fractions matched known proteins such as cytochrome c oxidase subunit-1, a protein associated with the complicated mitochondrial electron transport chain complex^34,35^. On the other hand, we also investigated how these peptide fractions exert their effects by measuring apoptotic and autophagy markers. Fractions 2 and 3 caused a reduction in the levels of the anti-apoptotic protein Bcl-2 in both HepG2 and HT-29 cells and an increase in the pro-apoptotic enzyme caspase-3, which suppresses cancer cell proliferation. Moreover, the downregulation of FGFR4 and F-19 genes may suggest that the treatment is promoting apoptosis, likely through the intrinsic (mitochondrial) pathway, rather than the extrinsic (Fas/Fas-L) pathway. Furthermore, the upregulated Beclin-1 levels in treated cells may indicate that autophagy pushes cells toward autophagy-mediated apoptosis rather than helping them survive. These results are in line with prior studies that point to an association of peptide-induced apoptosis and autophagy in the inhibition of cancer cell growth. AAP-H, a peptide derived from the sea anemone, *Anthopleura anjunae*, was shown to induce apoptosis in DU-145 human prostatic carcinoma cells via potential depolarization of the mitochondrial membrane and upregulation of cytochrome c, caspase-3, and caspase-9. Also, FIMGPY peptide obtained from skate cartilage induced apoptosis by upregulating the caspase-3 and changing the Bax/Bcl-2 ratio in HeLa cells^36^.

Our flow cytometry analysis demonstrated that the peptide fractions exhibited significant pro-apoptotic effects, as evidenced by an increase in both early and late apoptotic cells in treated HT-29 and HepG2 cell lines compared to untreated controls. This observation aligns with previous research indicating that marine-derived peptides can induce apoptosis in cancer cells by modulating key signaling pathways involved in cell survival and proliferation^37^. Additionally, cell cycle analysis revealed that fractions (2) and (3) induced G1/S phase arrest in HT-29 cells, suggesting that these peptides impede cell cycle progression, thereby inhibiting cellular proliferation. This finding is consistent with studies on marine-derived compounds, such as Didemnins, which have been shown to cause cell cycle arrest at the G1 phase by inhibiting protein synthesis necessary for DNA replication and repair^38^. Collectively, these results underscore the potential of marine-derived peptides as anticancer agents that exert their effects through the induction of apoptosis and disruption of cell cycle progression in cancer cells.

Moreover, examination of the treated cancer cells through morphological studies helped to understand the actions of the peptide fractions. HepG2 and HT-29 cells treated with fractions 2 and 3 had low nucleocytoplasmic ratios and the presence of more apoptotic bodies than the untreated control cells. These are changes in the cell’s surface morphology that are typical of apoptotic cells and support the biochemical and cytometric results.

## Conclusions

This study successfully identified and characterized bioactive peptides from *R. decussatus* with remarkable anticancer potential. These peptides exhibited selective cytotoxicity, effectively inducing apoptosis and autophagy in cancer cells while sparing normal cells. De novo sequence analysis in addition to anticancer scoring, exhibited that Pep25 was the most potent anticancer score (0.83). Flow cytometry analysis further confirmed their role in promoting early and late apoptosis and inducing G1/S phase cell cycle arrest, thereby inhibiting cancer cell proliferation and survival. These findings align with prior studies on marine-derived bioactive compounds, reinforcing their potential as a valuable source of novel anticancer agents.

Despite these promising in vitro results, comprehensive in vivo studies are essential to validate their efficacy, bioavailability, and safety profile. Further investigations into their molecular targets and signaling pathways will provide deeper insights into their mechanisms of action, facilitating their potential clinical translation. By advancing our understanding of these peptides, this research lays the foundation for the development of next-generation marine-derived therapeutics, offering new hope for more effective and targeted cancer treatments.

## Supplementary Information

Below is the link to the electronic supplementary material.

Supplementary Material 1

## Data Availability

The datasets used and/or analysed during the current study available from the corresponding author on reasonable request. The mass spectrometry proteomics data have been deposited to the ProteomeXchange Consortium via the PRIDE partner repository with the dataset identifier PXD067801 Submission details: Project Name: Novel peptides from the edible bivalve Ruditapes decussatus target apoptosis, autophagy, and FGF19-FGFR4 signaling in human cancer cell lines Project accession: PXD067801 Project DOI: Not applicable Reviewer access details Log in to the PRIDE website using the following details: Project accession: PXD067801 Token: HSSiTqxC5nxK Alternatively, reviewer can access the dataset by logging in to the PRIDE website using the following account details: Username: reviewer_pxd067801@ebi.ac.uk Password: kawMpEJFFGUq.

## References

[1] Sung, H. et al. Global cancer statistics 2020: GLOBOCAN estimates of incidence and mortality worldwide for 36 cancers in 185 countries. *CA. Cancer J. Clin.* **71**, 209–249 (2021). 33538338 10.3322/caac.21660

[2] World Health Organization. Cancer. World Health Organization. https://www.who.int/news-room/fact-sheets/detail/cancer (accessed 17 September 2025).

[3] Yang, S. et al. New insights into autophagy in hepatocellular carcinoma: Mechanisms and therapeutic strategies. *Am. J. Cancer Res.* **9**, 1329 (2019). 31392073 PMC6682711

[4] Kulik, L. & El-Serag, H. B. Epidemiology and management of hepatocellular carcinoma. *Gastroenterology* **156**, 477–491 (2019). 30367835 10.1053/j.gastro.2018.08.065 PMC6340716

[5] Chen, Q., Peng, K., Mo, P. & Yu, C. Histone demethylase JMJD2D: A novel player in colorectal and hepatocellular cancers. *Cancers* **14**, 2841 (2022). 35740507 10.3390/cancers14122841 PMC9221006

[6] Anand, U. et al. Cancer chemotherapy and beyond: Current status, drug candidates, associated risks and progress in targeted therapeutics. *Genes Dis.* **10**, 1367–1401 (2023). 37397557 10.1016/j.gendis.2022.02.007 PMC10310991

[7] Tie, J. et al. The potential of circulating tumor DNA (ctDNA) to guide adjuvant chemotherapy decision making in locally advanced rectal cancer (LARC). *J. Clin. Oncol.* **35**, 3521–3521 (2017).

[8] Ruiz-Torres, V. et al. An updated review on marine anticancer compounds: The use of virtual screening for the discovery of small-molecule cancer drugs. *Molecules* **22**, 1037 (2017). 28644406 10.3390/molecules22071037 PMC6152364

[9] Barreca, M. et al. Marine anticancer agents: An overview with a particular focus on their chemical classes. *Mar. Drugs* **18**, 619 (2020). 33291602 10.3390/md18120619 PMC7761941

[10] Wu, L., Ye, K., Jiang, S. & Zhou, G. Marine power on cancer: Drugs, lead compounds, and mechanisms. *Mar. Drugs* **19**, 488 (2021). 34564150 10.3390/md19090488 PMC8472172

[11] Hussein, A. A. A. et al. Identification and exploration of anticancer activity of novel peptides isolated from the edible bivalve *Callista chione* in hepatic and colon cancer cell lines. *Toxicol. Rep.* **14**, 101915 (2025). 39968051 10.1016/j.toxrep.2025.101915 PMC11833620

[12] Harnedy, P. A. & FitzGerald, R. J. Bioactive peptides from marine processing waste and shellfish: A review. *J. Funct. Foods* **4**, 6–24 (2012).

[13] Okasha, H. Fundamental uses of peptides as a new model in Both treatment and diagnosis. *Recent Pat. Biotechnol.* **18**, 110–127 (2024). 38282442 10.2174/1872208317666230512143508

[14] Gould, A. & Camarero, J. A. Cyclotides: Overview and biotechnological applications. *ChemBioChem* **18**, 1350–1363 (2017). 28544675 10.1002/cbic.201700153 PMC5812342

[15] Safavi-Hemami, H., Brogan, S. E. & Olivera, B. M. Pain therapeutics from cone snail venoms: From Ziconotide to novel non-opioid pathways. *J. Proteomics* **190**, 12–20 (2019). 29777871 10.1016/j.jprot.2018.05.009 PMC6214764

[16] Gogineni, V. & Hamann, M. T. Marine natural product peptides with therapeutic potential: Chemistry, biosynthesis, and pharmacology. *Biochim. Biophys. Acta BBA Gen. Subj.* **1862**, 81–196 (2018). 10.1016/j.bbagen.2017.08.014 PMC5918664 28844981

[17] Sridhar, K., Inbaraj, B. S. & Chen, B.-H. Recent developments on production, purification and biological activity of marine peptides. *Food Res. Int.* **147**, 110468 (2021). 34399466 10.1016/j.foodres.2021.110468

[18] Eghianruwa, Q. A., Osoniyi, O. R., Maina, N. & Wachira, S. Bioactive peptides from marine molluscs—A review. *Int. J. Biochem. Res. Rev.* **27**, 1–12 (2019).

[19] Macedo, M. W. F. S. et al. Marine organisms as a rich source of biologically active peptides. *Front. Mar. Sci.* **8**, 667764 (2021).

[20] Shahidi, F. & Saeid, A. Bioactivity of marine-derived peptides and proteins: A review. *Mar. Drugs* **23**, 157 (2025). 40278278 10.3390/md23040157 PMC12028762

[21] Dang, V. *Antiviral Immune Responses in Abalone and Influence of Potential Abiotic and Biotic Factors* (Flinders University of South Australia, 2012).

[22] Soliman, M. F. M., El-Shenawy, N. S., Tadros, M. M. & Abd El-Azeez, A. A. Impaired behavior and changes in some biochemical markers of bivalve (*Ruditapes decussatus*) due to zinc toxicity. *Toxicol. Environ. Chem.* **97**, 674–686 (2015).

[23] Pinto, A. F. M., Diedrich, J. K., Moresco, J. J. & Yates, J. R. Differential precipitation of proteins: A simple protein fractionation strategy to gain biological insights with proteomics. *J. Am. Soc. Mass Spectrom.* **34**, 2025–2033 (2023). 37527410 10.1021/jasms.3c00182

[24] Deyl, Z. Protein purification. Principles, high resolution methods and applications. *J. Chromatogr. B. Biomed. Sci. App.* **532**, 207–208 (1990).

[25] Perez-Riverol, Y. et al. The PRIDE database at 20 years: 2025 update. *Nucleic Acids Res.* **53**, D543–D553 (2025). 39494541 10.1093/nar/gkae1011 PMC11701690

[26] Ramarathinam, S. H. *et al.**A Peptide-Signal Amplification Strategy for the Detection and Validation of Neoepitope Presentation on Cancer Biopsies*. pp. 2020–06 (2020).

[27] Agrawal, P., Bhagat, D., Mahalwal, M., Sharma, N. & Raghava, G. P. S. AntiCP 2.0: An updated model for predicting anticancer peptides. *Brief. Bioinform.* **22**, 153 (2021). 10.1093/bib/bbaa153 32770192

[28] Okasha, H., Nasr, S. M. & Samir, S. Recombinant expression of Cec-B peptide in *Escherichia coli* with a significant anticancer effect on hepatocellular carcinoma. *Curr. Pharm. Biotechnol.* **22**, 1235–1245 (2021). 33397234 10.2174/1389201022666210104121709

[29] Zhang, Y., Wang, C., Zhang, W. & Li, X. Bioactive peptides for anticancer therapies. *Biomater. Transl.* **4**, 5 (2023). 37206303 10.12336/biomatertransl.2023.01.003 PMC10189813

[30] Wang, L., Dong, C., Li, X., Han, W. & Su, X. Anticancer potential of bioactive peptides from animal sources. *Oncol. Rep.* **38**, 637–651 (2017). 28677775 10.3892/or.2017.5778

[31] Suarez-Jimenez, G.-M., Burgos-Hernandez, A. & Ezquerra-Brauer, J.-M. Bioactive peptides and depsipeptides with anticancer potential: Sources from marine animals. *Mar. Drugs* **10**, 963–986 (2012). 22822350 10.3390/md10050963 PMC3397454

[32] Kamalakkannan, P. Marine sponges a good source of bioactive compounds in anticancer agents. *Int. J. Pharm. Sci. Rev. Res.* **31**, 132–135 (2015).

[33] Librizzi, M. et al. Natural anticancer peptides from marine animal species: Evidence from in vitro cell model systems. *Cancers* **16**, 36 (2023). 38201464 10.3390/cancers16010036 PMC10777987

[34] Hamza-Chaffai, A., Amiard, J. C. & Cosson, R. P. Relationship between metallothioneins and metals in a natural population of the clam *Ruditapes decussatus* from Sfax coast: a non-linear model using Box-Cox transformation. *Comp. Biochem. Physiol. C Pharmacol. Toxicol. Endocrinol.* **123**, 153–163 (1999). 10442824 10.1016/s0742-8413(99)00023-7

[35] Sekine, Y., Yamakawa, H., Takazawa, S., Lin, Y. & Toba, M. Geographic variation of the COX1 gene of the short-neck clam *Ruditapes philippinarum* in coastal regions of Japan and China. *Venus J. Malacol. Soc. Jpn.* **65**, 229–240 (2006).

[36] Zhang, Q.-T. et al. Recent advances in small peptides of marine origin in cancer therapy. *Mar. Drugs* **19**, 115 (2021). 33669851 10.3390/md19020115 PMC7923226

[37] Magiatis, N. Role of marine-derived peptides in anticancer therapy. *J. Pharmacogn. Nat. Prod.* **10**, 335 (2024).

[38] Ali, M. A. et al. Didemnins as marine-derived anticancer agents: Mechanistic insights and clinical potential. *Med. Oncol.* **42**, 43 (2025). 39797969 10.1007/s12032-024-02594-0

