# Supplementary material for: Novel peptides from the edible bivalve Ruditapes decussatus target apoptosis, autophagy, and FGF19-FGFR4 signaling in human cancer cell lines
Source: Sci Rep. 2025 Oct 1;15:34283. doi: 10.1038/s41598-025-20094-7 (PMC12489141; doi:10.1038/s41598-025-20094-7)
Supplement: Supplementary file 1 — Supplementary Material 1 [file 41598_2025_20094_MOESM1_ESM.docx]

**Novel peptides from the edible bivalve *Ruditapes decussatus* target apoptosis, autophagy, and FGF19-FGFR4 signaling in human cancer cell lines**

Ahmed A.A. Hussein^a^, Maha B. Salem ^b^ , Mohamed ElZallat^c^, Samah I. Ghoname^a^, Mohamed R. Habib^a^, Olfat Ali Hammam ^d^, Ehab El-Dabaa^e^ , and Hend Okasha^e^

*Departments of Medical Malacology^a^, Pharmacology^b^, ^c^Immunology^c^, Pathology^d^, and Biochemistry and Molecular Biology^e^, Theodor Bilharz Research Institute, Warrak El-Hadar, Imbaba, Giza 12411, Egypt.*

**Affiliations:**

1. **Ahmed A.A. Hussein (Corresponding author)**

Researcher, Medical Malacology Department, Theodor Bilharz Research Institute, Giza, Egypt

e-mail: [ahmed.abdelazeez@science.suez.edu.eg](mailto:ahmed.abdelazeez@science.suez.edu.eg)

ORCID: 0000-0002-1427-1026

1. **Maha B. Salem**

Researcher, Pharmacology Department, Theodor Bilharz Research Institute, Giza, Egypt

e-mail: [drmahabadr86@gmail.com](mailto:drmahabadr86@gmail.com)

ORCID: 0000-0003-2806-480X

1. **Mohamed ElZallat**

Researcher, Immunology Department, Theodor Bilharz Research Institute, Giza, Egypt

e-mail: [zallatzallat@gmail.com](mailto:zallatzallat@gmail.com)

1. **Samah Ibrahim Ghoname**

Associate Professor, Medical Malacology Department, Theodor Bilharz Research Institute, Giza, Egypt

e-mail: [samahghoname@gmail.com](mailto:samahghoname@gmail.com)

1. **Mohamed Ramadan Habib**

Associate Professor, Medical Malacology Department, Theodor Bilharz Research Institute, Giza, Egypt

e-mail: [m.habib@tbri.gov.eg](mailto:m.habib@tbri.gov.eg)

ORCID: 0000-0003-0820-9290

1. **Olfat Ali Hammam**

Professor, Pathology Department, Theodor Bilharz Research Institute, Giza, Egypt

email: [totoali1.@hotmail.com](mailto:totoali1.@hotmail.com)

ORCID: [0000-0002-4965-5804](https://orcid.org/0000-0002-4965-5804)

1. **Ehab El-Dabaa**

Professor, Head of Center of Excellence in Recombinant Biopharmaceutical Proteins, Biochemistry and Molecular Biology Department, Theodor Bilharz Research Institute, Giza, Egypt

e-mail: [ehabphd@yahoo.com](mailto:ehabphd@yahoo.com)

1. **Hend Okasha**

Associate Professor, Biochemistry and Molecular Biology Department, Theodor Bilharz Research Institute, Giza, Egypt

e-mail: [hend.oaa@gmail.com](mailto:hend.oaa@gmail.com)

ORCID: 0000-0003-1125-245X

**Table. S1: Analysis of peptides in fraction (2) using novor.cloud**

| **No.** | **m/z** | **z** | **Peptide Mass** | **Error (ppm)** | **Length** | **De Novo Peptide** |
| --- | --- | --- | --- | --- | --- | --- |
|  | 1662.104 | 4 | 6644.307 | 12.2 | 53 | DVKKLLLRMDWRHKKWWYMM(O)DDCDDDDDCDDNCNM(O)M(O)YCM(O)CGECM(O)M(O)CCCDMDDD |
|  | 1944.168 | 3 | 5829.524 | -7 | 42 | DPPDWWWWWSAPAWYYM(O)FFEDYYWM(O)MDCRRRRFRRSPKM(O)HMN |
|  | 1916.878 | 3 | 5747.529 | 14.3 | 43 | KAARRRRRRAWWRTWM(O)M(O)M(O)M(O)DNCCCCCCCYWCNWKKKRRRSYNE |
|  | 1911.523 | 3 | 5731.485 | 10.8 | 42 | LLVPWWRWRRRRRADMEGYPVRHKKKKKLRRRRRRRKLKRRL |
|  | 1873.03 | 3 | 5616.147 | -13.9 | 43 | DAHTWM(O)M(O)M(O)M(O)AM(O)M(O)M(O)M(O)M(O)M(O)M(O)AFGALLLNWWWWWEGPVNENYM(O)NYEN |
|  | 1807.414 | 3 | 5419.268 | -8.5 | 42 | M(O)DDDMVWPLRRRRRKTARLRFKLM(O)M(O)M(O)M(O)M(O)CCCNM(O)ECM(O)MVHCCM |
|  | 1766.697 | 3 | 5296.998 | 13.6 | 40 | DWRLLLLRDPYLKKKEKFWWEEEFKKKKKKVRKKSKLM(O)M(O)Q |
|  | 1753.395 | 3 | 5257.233 | -13.4 | 38 | DEQWWM(O)EDDWWWWRRWYM(O)SSLWMAMM(O)M(O)GVM(O)RRAVKYMQ |
|  | 1732.144 | 3 | 5193.48 | -13.5 | 33 | DRHYYNWWWWWWEWWWWLWRRRRRRRRFHDMCR |
|  | 1732.07 | 3 | 5193.122 | 12.6 | 41 | DAHTAGKKLDRRRLLMCQWWWWYM(O)M(O)YQYM(O)M(O)CCGGCGMDM(O)MQ |
|  | 1713.596 | 3 | 5137.74 | 5.3 | 35 | HSKWWWWWWWRKVGVLVLVLWYWRKDWRRRLWWWK |
|  | 1653.281 | 3 | 4956.821 | 0.2 | 38 | DHTARKKVLRRKKKLEGGEWWWYM(O)RKRKVLRKKKLRM(O)C |
|  | 1647.117 | 3 | 4938.145 | 37 | 35 | YLLRRRRYSVRRRRLLLRRRKTLKLHRRRRRRRRR |
|  | 1645.756 | 3 | 4934.317 | -14.3 | 42 | DATNSEDDDDDCCCCCQWYMEVM(O)EDCCCM(O)DMECHCM(O)CCYMCG |
|  | 1608.391 | 3 | 4822.219 | -13.7 | 31 | M(O)RHVSRRRKHRRAQWWWWWWWWWWWWM(O)MM(O)QM(O) |
|  | 1586.385 | 3 | 4756.072 | 13.1 | 34 | RLPRCHRKRKLLVWWYYM(O)M(O)RM(O)M(O)EENWM(O)M(O)M(O)CM(O)MQM |
|  | 1581.349 | 3 | 4741.017 | 2.1 | 33 | DARRRRRGRVM(O)CSSSWWWWM(O)DRM(O)WWWWWM(O)M(O)M(O)DH |
|  | 1561.624 | 3 | 4681.878 | -6.3 | 37 | DYYM(O)ESKNVAGLLACGNCSCWM(O)WM(O)M(O)GCYRREWWWRM(O)S |
|  | 1560.027 | 3 | 4677.042 | 3.4 | 34 | DECM(O)WWM(O)MCWYEGEHRVYKKRRLSWWWYRREM(O)GG |
|  | 1537.519 | 3 | 4609.581 | -10.2 | 40 | DVPPHAECM(O)CCCCCDCCDCCDCDM(O)ACYCRRRLEPLHAWFG |
|  | 1529.608 | 3 | 4585.862 | -13.2 | 34 | DAHTWWWPHVRYYDM(O)M(O)M(O)M(O)DWMQNDWRVGHHVM(O)QM |
|  | 1500.771 | 3 | 4499.359 | -14.9 | 34 | DRNCNWWRKKLRMRKRRKRKKKKKTKEDDMM(O)GCM(O) |
|  | 1492.467 | 3 | 4474.36 | 4 | 36 | DAHTRKKKKALVDWWADWYFQYFDRKKKVALKPGDH |
|  | 1484.96 | 3 | 4451.822 | 8.5 | 32 | RLCRTWWWSPKWWWWYWFQQCSVM(O)M(O)DCDDM(O)MQ |
|  | 1480.152 | 3 | 4437.396 | 8.8 | 31 | KLAHRRRSKPKKWWWQGARNWWRWWWRRRFF |
|  | 1478.401 | 3 | 4432.172 | 2.4 | 34 | LLLRVSFKNRRVHKEWDDNWRRRRRPMMCCMDDD |
|  | 1478.096 | 3 | 4431.3 | -7.4 | 33 | DAHTWWWHNVWWWWRRRRPRRKKTGVRRAFMAG |
|  | 1469.135 | 3 | 4404.424 | -9.6 | 32 | DWAWWWM(O)M(O)TQWWADWYYM(O)M(O)M(O)M(O)CCCCYCCAM(O)M(O)Q |
|  | 1457.357 | 3 | 4369.16 | -25.2 | 40 | Q(Pyro-Glu)M(O)CCNYM(O)CCCAGCCFQRHAVCCCCRGCCSCCCCDECECGD |
|  | 1447.285 | 3 | 4338.777 | 13.2 | 36 | LAM(O)KKKKKRRRTALPPLRLRRLGATAKKRRRKKKGQ |
|  | 1439.156 | 3 | 4314.462 | -3.6 | 34 | DAWRRRKVSRQWWKFPKKLLPLHLQLKLKPGRM(O)C |
|  | 1431.27 | 3 | 4290.813 | -5.7 | 35 | DCCCCCNCM(O)YCDTLWYRSPKMNFKRRKFRPVM(O)TTG |
|  | 1423.551 | 3 | 4267.675 | -10.6 | 31 | DASSWWWRELWWWWWWWWM(O)TEDCM(O)MNDGGNN |
|  | 1421.976 | 3 | 4262.844 | 14.9 | 34 | LFKKKRKRKVHTGRRRKLLLLSVRRKLLLLRVVR |
|  | 1421.618 | 3 | 4261.879 | -10.8 | 32 | DCACCCWCM(O)M(O)ERMKRRRALKRRFLGEWWWM(O)HH |
|  | 1414.424 | 3 | 4240.2 | 12.1 | 31 | RLRPLKSWWAMDVSM(O)M(O)LRRRRLPLWWWWM(O)HL |
|  | 1409.532 | 3 | 4225.636 | -14.4 | 33 | DM(O)MCCCCM(O)CAYECCCCCFFRRRRVHCRRQYRTR |
|  | 1409.507 | 3 | 4225.444 | 13 | 31 | FRPKRRRKGEQNM(O)RRFFLKRLRRLRFKKFQM |
|  | 1409.491 | 3 | 4225.404 | 11.6 | 34 | LLLM(O)RADGEMEM(O)M(O)M(O)CWKLPKKKKKKKKKKKKAKK |
|  | 1399.377 | 3 | 4195.07 | 9.1 | 30 | DNPNVGRKSQWWWWRRRQLWYRRHEKYFMM |
|  | 1392.418 | 3 | 4174.197 | 8.6 | 37 | DACCGWM(O)CGPPSGEWWDNFCM(O)CCDQNHCCCCCGEECD |
|  | 1390.924 | 3 | 4169.694 | 13.2 | 31 | DTRRKVRRRRRKVFM(O)M(O)EMCCCCM(O)M(O)M(O)M(O)M(O)M(O)MDM(O) |
|  | 1365.363 | 3 | 4093.048 | 4.5 | 33 | KLLLRRLTHCCGCCSWVSLPHWWWFERRNLPPT |
|  | 1349.821 | 3 | 4046.412 | 7.2 | 34 | DPPDCGRRVVKPLRRQKRKKKLVKKKKRLSGADY |
|  | 1346.828 | 3 | 4037.522 | -14.6 | 28 | DDCNM(O)M(O)SSDHQWWWWWFERRWRM(O)M(O)M(O)M(O)QM(O) |
|  | 1344.51 | 3 | 4030.552 | -10.7 | 31 | DYECHFGDLFWQRRRRRHM(O)M(O)DCHCCCCCM(O)DK |
|  | 1344.086 | 3 | 4029.194 | 10.2 | 33 | DPPDWHTWGAPSFERRKLLLLVPRLRRRRPHDG |
|  | 1321.981 | 3 | 3962.978 | -14.4 | 32 | DMCCCCCM(O)CYYWERYKKKKGTRKKLLLLLLVL |
|  | 1321.984 | 3 | 3962.975 | -11.2 | 27 | DPPDRHFWRRMRRRRRRWWQWRHEYNE |
|  | 1321.984 | 3 | 3962.887 | 10.7 | 31 | DAHNGGGRVWMWWVSRRYFFHHEKRVRLM(O)MC |
|  | 1321.987 | 3 | 3962.886 | 13.1 | 30 | HPTM(O)GM(O)HCRCEWM(O)WLAKRRRRNWPQKRTTY |
|  | 1321.985 | 3 | 3962.884 | 12.6 | 31 | KLAKKKKKKGVLGEM(O)M(O)HCREWYYYFQM(O)DDMM |
|  | 1321.65 | 3 | 3961.976 | -12 | 31 | DACAAKRFYM(O)M(O)M(O)M(O)NWYM(O)RM(O)NRKKLLLLLLVL |
|  | 1321.645 | 3 | 3961.861 | 13.1 | 28 | DAKADKKDRRLWWWWWKNRYYYYKM(O)EDM(O) |
|  | 1321.581 | 3 | 3961.76 | -10 | 33 | DDPLCCCCM(O)M(O)M(O)DM(O)KKKKKKLRLM(O)VSGSEPKM(O)MQ |
|  | 1321.314 | 3 | 3960.96 | -10.3 | 30 | DALAKRLQNWWWWWVSWHEREENSYKKKTT |
|  | 1319.302 | 3 | 3954.845 | 9.5 | 29 | DAWKFKRRRRRLMNWWWWM(O)ESERM(O)HADGC |
|  | 1304.648 | 3 | 3910.864 | 14.7 | 30 | EYGVWWKQRAM(O)M(O)RDWFYENFLLLVPPVYNE |
|  | 1301.993 | 3 | 3902.968 | -3 | 34 | LLRGAKYWCLPM(O)NCGHLLLPGGVRLLLYYTM(O)GFC |
|  | 1952.315 | 2 | 3902.654 | -10 | 33 | DVPPWLPYKQGYECCCDEAM(O)M(O)MYLLSWLPM(O)VSG |
|  | 1299.487 | 3 | 3895.49 | -13.3 | 28 | DAM(O)DCM(O)MDYMWWWM(O)M(O)M(O)M(O)WCM(O)RCREKKKK |
|  | 1294.561 | 3 | 3880.712 | -13.2 | 30 | DYNPPMQRRRRMLRKLARNWM(O)MM(O)EDM(O)DCCS |
|  | 1906.926 | 2 | 3811.885 | -12.5 | 30 | DVLPKKKSVYMQM(O)NVKM(O)DNNYYLQRRLYEN |
|  | 1266.536 | 3 | 3796.599 | -2.9 | 32 | DAHTWSHDM(O)LLTDCGM(O)CCM(O)DFAKKLVVWSFQM |
|  | 1897.768 | 2 | 3793.468 | 14.2 | 34 | DVHGRFAGKKKHSVDHVHDNCNSCCGM(O)DCCMDDD |
|  | 1897.091 | 2 | 3792.122 | 12.2 | 30 | FKKLARKLKKVKKWWWEGYYM(O)RELPLAPPS |
|  | 1260.379 | 3 | 3778.08 | 9.5 | 27 | FLVVLKKWWWWWWWWWVSYVRVGKLKK |
|  | 1225.198 | 3 | 3672.6 | -7.2 | 30 | DHARRHLLLLRRKVYCDDCDMEEYGDM(O)DCS |
|  | 1223.464 | 3 | 3667.339 | 8.7 | 33 | DACCNSVVRFARKSPHEGM(O)HM(O)HCCNCCSGM(O)M(O)CG |
|  | 1833.065 | 2 | 3664.135 | -5.4 | 27 | DARRRLKLARLWFKRRRKWWFRRVPPD |
|  | 1221.986 | 3 | 3662.989 | -14.3 | 27 | DWFDFHKKKKKRAKLRRDWEGWWYKLL |
|  | 1221.986 | 3 | 3662.988 | -13.8 | 31 | DAWNKPWEDDTWCCCACFCCCCCCCCM(O)M(O)CCM(O) |
|  | 1221.989 | 3 | 3662.979 | -9.1 | 29 | RLPPWRRRKKKKLRRYRCSHM(O)CSVQAGNH |
|  | 1221.988 | 3 | 3662.888 | 14.9 | 27 | DAVLGKQWDAWWWWWWLRRYYQKKVPL |
|  | 1221.876 | 3 | 3662.561 | 12.6 | 28 | DAHCPPRYWQAVWWRRM(O)QYHMYM(O)GGRCM(O) |
|  | 1831.319 | 2 | 3660.588 | 10 | 29 | DAMVPPVMECGGWWWWMKRQWRM(O)EKM(O)CTA |
|  | 1821.566 | 2 | 3641.072 | 12.2 | 26 | LLPRPLWWWRYGTHLWRRRRRRRLQQ |
|  | 1809.974 | 2 | 3617.936 | -0.5 | 26 | DVPEHWRRRRRRRM(O)KVEGRRRRHMYQ |
|  | 1808.938 | 2 | 3615.897 | -10 | 31 | DHCCCCSPNM(O)ECCEQHDCDM(O)ECCCMCMDMDD |
|  | 1806.856 | 2 | 3611.645 | 14.2 | 29 | DNPGGWKKKKKRGPTGRLM(O)EM(O)M(O)M(O)RM(O)M(O)M(O)QM(O) |
|  | 1803.124 | 2 | 3604.285 | -14.2 | 27 | DVPLWWWM(O)M(O)MTM(O)MCCCWEGWM(O)EYSRCM(O) |
|  | 1800.819 | 2 | 3599.672 | -13.4 | 29 | MMEPHMCCM(O)M(O)CEAKLWKKKKKWHKLM(O)TAA |
|  | 1799.48 | 2 | 3596.906 | 11 | 29 | GGYM(O)HKKLKARQWWGGGLLHKLLRWRM(O)MQ |
|  | 1798.386 | 2 | 3594.775 | -4.7 | 26 | DVGHLLPWWWWWM(O)WHYKRRQPLLM(O)KD |
|  | 1783.946 | 2 | 3565.907 | -8.4 | 26 | DAWNRKKYKSLVWHHKWLYKKWYM(O)KK |
|  | 1184.583 | 3 | 3550.676 | 14.2 | 26 | M(O)EGADHKKYRRRFRRRLPWWWHM(O)CCD |
|  | 1181.714 | 3 | 3542.076 | 12 | 28 | DFCPEWM(O)CCCCCM(O)M(O)M(O)M(O)EWWM(O)DCGQCSVR |
|  | 1761.303 | 2 | 3520.54 | 14.9 | 27 | DARFMRWPKM(O)HECCM(O)M(O)CEPKRRLMPYQ |
|  | 1174.4 | 3 | 3520.138 | 11.4 | 26 | KLASLVLYLLWWRWRFFKWRKKKLKK |
|  | 1167.052 | 3 | 3498.187 | -14.7 | 29 | DMHDMCM(O)DWRRRHVLM(O)EDDCCCCCSSDGD |
|  | 1748.429 | 2 | 3494.885 | -11.7 | 32 | DMDCNCCCGGM(O)CCTTTMVM(O)ECCCCCCCEGRM(O)C |
|  | 1744.312 | 2 | 3486.656 | -13.7 | 26 | DM(O)M(O)M(O)M(O)DM(O)DHAKAKKYFKRRRRKKYDQ |
|  | 1729.156 | 2 | 3456.308 | -2.9 | 27 | DHDCNWWWM(O)M(O)KKTLLYM(O)M(O)M(O)M(O)M(O)CCGGSL |
|  | 1725.057 | 2 | 3448.063 | 10.7 | 30 | DAMNCQM(O)WQWDTGCGSEGCCCEGQYM(O)M(O)QCC |
|  | 1706.866 | 2 | 3411.692 | 7.6 | 24 | DVM(O)VWRLHKWWFM(O)ERVWWKKKRM(O)C |
|  | 1701.625 | 2 | 3401.211 | 7 | 29 | DGHDM(O)NCCCCCCM(O)M(O)CVSGVQSRRFFLEYN |
|  | 1696.583 | 2 | 3391.109 | 12.4 | 31 | SMNCDNSWQGGSDM(O)DDHNDNSNCPDNSEGQC |
|  | 1122.33 | 3 | 3364 | -9.1 | 28 | DAPQRRRRLGVKQFVSHLAKKKKFPKFV |
|  | 1677.7 | 2 | 3353.43 | -13.3 | 27 | DACCM(O)M(O)M(O)EWWWACFADPKLPKRVVFEN |
|  | 1662.591 | 2 | 3323.123 | 13.6 | 27 | LLPQRKKKKKAALGRRRKWRRRRAGPL |
|  | 1650.737 | 2 | 3299.447 | 3.9 | 27 | DPPDM(O)M(O)M(O)MNM(O)M(O)MDKKKKKKKRGDM(O)GMA |
|  | 1647.152 | 2 | 3292.249 | 12 | 25 | DAWYM(O)FYM(O)RTGSSM(O)M(O)CKWVSYYM(O)M(O)E |
|  | 1645.795 | 2 | 3289.535 | 12.2 | 24 | LLLLQSKPRQWWWWWWYMMMM(O)GM(O)A |
|  | 1645.546 | 2 | 3289.097 | -5.6 | 27 | DMDEMDDHHDCWWWFHHGGAEGEDDDC |
|  | 1640.395 | 2 | 3278.823 | -14.4 | 27 | AAPNSWFERKLARLRRKKKRAFKM(O)GM(O)A |
|  | 1635.385 | 2 | 3268.731 | 7.6 | 28 | SCSYRNATKLRQLLVGELLFKKLLEECD |
|  | 1086.844 | 3 | 3257.504 | 1.8 | 24 | DAWWWNVQWYWEGYGERNRLLRM(O)Q |
|  | 1079.734 | 3 | 3236.133 | 14.5 | 27 | RLRKLGVKKLPKKKKKKKKGEWKPPPL |
|  | 1615.183 | 2 | 3228.32 | 9.9 | 24 | GYGM(O)EENWM(O)M(O)M(O)M(O)EDWRRHKLLCM(O)R |
|  | 1613.012 | 2 | 3223.979 | 9.5 | 25 | DAYYM(O)WYM(O)M(O)M(O)M(O)CCCGEM(O)GNEM(O)NRCM(O) |
|  | 1612.954 | 2 | 3223.935 | -13.2 | 24 | DM(O)ECWWWYYM(O)SM(O)M(O)M(O)M(O)M(O)DDCM(O)AGCM(O) |
|  | 1612.01 | 2 | 3222.028 | -7 | 25 | DMGCDFM(O)M(O)WWYYGEERQM(O)DCEECCD |
|  | 1608.382 | 2 | 3214.785 | -11.1 | 24 | M(O)EDYM(O)M(O)M(O)M(O)M(O)M(O)M(O)M(O)M(O)CM(O)DDDM(O)CCCHM |
|  | 1069.577 | 3 | 3205.742 | -10.6 | 26 | ELTHFGLKPGWRRRQQFRMRPRLPSG |
|  | 1069.455 | 3 | 3205.387 | -13.2 | 23 | M(O)M(O)M(O)CCCCHWFAMWFRREKYRRKR |
|  | 1063.037 | 3 | 3186.081 | 2.9 | 25 | M(O)ENCCMDQWM(O)M(O)MCMCCCRRVPLM(O)M(O)Q |
|  | 1592.838 | 2 | 3183.626 | 11.3 | 24 | FVGRRRRRKRKKTYTFEYFNEECD |
|  | 1592.803 | 2 | 3183.545 | 14.8 | 28 | DAM(O)HQPSKKLGM(O)ELTLLLHLLCPACMQM |
|  | 1592.088 | 2 | 3182.209 | -14.9 | 24 | DPPDYKTKGAWWFM(O)HM(O)M(O)M(O)M(O)M(O)M(O)RM(O)C |
|  | 1590.784 | 2 | 3179.598 | -14.2 | 26 | DAHTYFNWGGPDVSKKRRRRKSLM(O)MQ |
|  | 1058.851 | 3 | 3173.486 | 14.7 | 27 | DAVTYDDPRRHYCKKGVRHGPLHYMCG |
|  | 1581.125 | 2 | 3160.258 | -7.1 | 27 | DGMHECCCCCCCCWRFKKKRALLSGYD |
|  | 1568.978 | 2 | 3135.978 | -11.8 | 26 | DDNQM(O)HCCRMMAEKCCCRHM(O)CCM(O)M(O)CG |
|  | 1558.64 | 2 | 3115.244 | 7 | 26 | KKKKKKMWRHEM(O)CCCCCCDCPSM(O)M(O)GA |
|  | 1036.729 | 3 | 3107.195 | -10 | 26 | AGHEFKKKWWWEGM(O)ECNEDCAAQCCC |
|  | 1545.952 | 2 | 3089.843 | 15 | 24 | RLPLMHKKLTKKARRRRRARLM(O)QM |
|  | 1543.03 | 2 | 3084.007 | 12.8 | 25 | GGYM(O)WM(O)DM(O)M(O)M(O)WM(O)M(O)M(O)EESSESAPHGD |
|  | 1539.473 | 2 | 3076.957 | -8.1 | 26 | NYM(O)M(O)QNQEDKTCCCCCGCCEMEAYNE |
|  | 1531.951 | 2 | 3061.844 | 14.6 | 24 | WLLVRRKFKLKKKLRKKLM(O)QM(O)AMG |
|  | 1524.009 | 2 | 3045.965 | 12.6 | 25 | M(O)YGM(O)M(O)M(O)EGEGKYDM(O)EM(O)M(O)CCDNSFAM |
|  | 1519.675 | 2 | 3037.295 | 12.9 | 24 | KLAHWWLHSHM(O)AQM(O)WELYM(O)M(O)AGNC |
|  | 1519.146 | 2 | 3036.319 | -13.5 | 24 | DMPPRRRRHVCCSWWVSWEEM(O)CTA |
|  | 1518.511 | 2 | 3034.965 | 14.2 | 24 | KLLVLKRRRKKVRRRRAKRLPGHD |
|  | 1509.979 | 2 | 3017.899 | 14.8 | 26 | DARM(O)ERLLWM(O)DDCM(O)DCCCCCCCSGCC |
|  | 1505.531 | 2 | 3009.09 | -14.2 | 24 | DCCCCRM(O)CCMMCCGERRRRRNEYN |

(O): methionine oxidation
